# 18F-FDG PET/CT metabolic network connectivity in clinical staging of diffuse large B-cell lymphoma

**DOI:** 10.3389/fonc.2026.1856758

**Published:** 2026-08-24

**Authors:** Jinhai Zou, Han Zou, Chang Liu, Chen Liang, Yufei Liang, Meiyu Zheng, Chunying Li

**Affiliations:** 1 Department of Nuclear Medicine, Cangzhou Central Hospital, Cangzhou, Hebei, China; 2 Second Clinical Medical College of Lanzhou University, Lanzhou, Gansu, China; 3 Department of Gastroenterology III, Cangzhou Central Hospital, Cangzhou, Hebei, China

**Keywords:** 18F-FDG PET/CT, diffuse large B-cell lymphoma, lymphoma clinical stage, lymphoma diffusion parameters, metabolic network connectivity

## Abstract

**Introduction:**

Diffuse large B-cell lymphoma (DLBCL) is the most common and highly heterogeneous subtype of non-Hodgkin lymphoma, and accurate staging is essential for treatment planning and prognosis. This study explored the value of 2-[18F]fluoro-2-deoxy-D-glucose positron emission tomography/computed tomography (18F-FDG PET/CT) metabolic network connectivity for the clinical staging of DLBCL.

**Methods:**

Forty-two previously untreated patients (23 males, 19 females; aged 30-82 years) diagnosed as *de novo* DLBCL, not otherwise specified, were retrospectively analyzed (stage I, n=3; II, n=5; III, n=9; IV, n=25). Lesions were automatically delineated using a 41% SUVmax threshold. Four metabolic and four diffusion parameters were computed, and a radiomics-based lesion metabolic network was constructed for each patient with stage II-IV disease.

**Results:**

The degree Pearson correlation coefficient (DPCC) of the metabolic network differed significantly among stages II-IV (n=39, p<0.001) and between stage II (n=5) and stages III+IV (n=34) (p<0.001), whereas conventional metabolic, diffusion, and other network parameters showed no significant differences. DPCC decreased with increasing stage, indicating rising inter-lesion metabolic heterogeneity.

**Discussion:**

The DPCC of 18F-FDG PET/CT metabolic network connectivity is associated with clinical stage in DLBCL patients with multiple measurable lesions and may provide complementary network-level information. These exploratory findings require external validation in larger, multicenter cohorts.

## 1 Introduction

Lymphomas are a group of highly heterogeneous malignancies of the lymphatic system, primarily categorized into Hodgkin lymphoma (HL) and non-Hodgkin lymphoma (NHL). Diffuse large B-cell lymphoma (DLBCL) is the most common type of non-Hodgkin lymphoma, accounting for approximately 30% of all NHL and over 40% in Asian countries (1). Diagnosing DLBCL mainly relies on pathological examination, including morphologic assessments of tumor tissue and immunohistochemical analysis (2). It is crucial to identify clinical parameters and biomarkers that aid in the early diagnosis and accurate staging of DLBCL patients (3).

In recent years, 18F-FDG PET/CT whole-body scans have become valuable tools for detecting multiple lesions and assessing the metabolic activity of tumors in lymphoma patients. These scans assist physicians in quantitatively evaluating the extent of tumors and outcomes of treatment. The clinical importance of 18F-FDG PET/CT in the early diagnosis, clinical staging, efficacy evaluation, and prognosis of DLBCL have been established. However, common quantitative parameters of PET, such as standardized uptake value (SUV), total metabolic tumor volume (TMTV), and total lesion glycolysis (TLG), do not adequately reflect the spatial distribution of lymphoma tumors (4–7) and have limited utility in clinical staging. Recently introduced FDG PET/CT diffusion parameters, including the largest distance between two lesions (Dmax), the distance between the largest lesion and the lesion farthest from that bulk (Dmaxbulk), the sum of the distances of the bulky lesion from all other lesions (SPREADbulk), and the largest value, over all lesions, of the sum of the distances from a lesion to all the others (SPREAD), can provide lymphoma dissemination properties and have been used to predict treatment efficacies of lymphoma (8–10). A recent multidisciplinary expert consensus further highlighted the value of baseline PET/CT-derived quantitative parameters, including MTV, TLG, and Dmax, for risk stratification in DLBCL (11). Nonetheless, these diffusion parameters do not capture the intrinsic relationships of metabolic network connectivity between lymphoma lesions.

Graph theory is a branch of mathematics that studies graphs, which consist of a number of nodes (also called vertices) and connections (known as edges) between them. Network connectomics applies graph theory to the construction and analysis of complex networks, such as the neuronal system. By mapping and examining the network connections among tissues or lesions, researchers can gain a deeper understanding of tissue structures, the spatial distribution of lesions, and their intrinsic relationships. This analysis provides valuable insights for both clinical and scientific research. 18F-FDG PET brain metabolic network connectivity has proven to be highly valuable in the analysis and understanding of brain diseases, including cognitive impairment and Parkinson’s disease (12, 13). Constructing an individual brain metabolic network or metabolic network between tissues and organs typically requires time series data, that is, dynamic scanning data. However, the continuous acquisition of data over long periods poses challenges for the clinical application of dynamic scanning. Zhao et al. introduced a regional radiomics similarity networks (R2SN) to study the relationships between different brain regions (14). They verified this method is both credible and reproducible. By combining radiomics and graph theory, the R2SNs was constructed using radiomics features from various brain regions based on MRI images. Graph-theoretic parameters were then calculated to investigate the inner relationship of the brain network.

In oncology studies, tumor regions, cell populations, primary lesions, and metastatic lesions can be viewed as nodes within a network. The relationships between these nodes, such as spatial proximity, biological similarity, or metabolic interactions, can be represented by edges (15). Based on previous work by Zhao et al., we hypothesized the R2SNs can also be applied to oncology studies for the quantitative analysis of tumor locations, as well as the intricate relationships and interactions within tumors. There have been reports that advanced non-small cell lung cancer can affect brain network connectivity (16). Liu et al. reported that DLBCL patients showed changes compared to normal controls, and there were also changes after treatment (17). However, there have been no reports on brain metabolic network connectivity between different stages of lymphoma. Moreover, there is a lack of quantitative analysis on brain network connections in the literature, such as the shortest path, degree, and correlation of brain region connections. In addition, the R2SNs method is the latest feature extraction method, which extracts more radiomics parameters to construct brain networks than Kullback Leibler divergence based similarity (KLS).

In this study, we aim to explore the feasibility and value of 18F-FDG PET/CT metabolic network connectivity for clinical staging of DLBCL by analyzing and comparing conventional metabolic parameters, diffusion parameters, and metabolic network parameters.

## 2 Materials and methods

In this retrospective study, we randomly collected 42 patients (23 males and 19 females, aged 30 to 82 years) diagnosed with DLBCL through histopathologic diagnosis at the Cangzhou Central Hospital in Hebei, China, from January 2021 to December 2023. All cases were diagnosed as *de novo* DLBCL, not otherwise specified (NOS), based on the available histopathological and immunohistochemical data according to the 5th edition of the World Health Organization classification of hematolymphoid tumors (18). Cases with pathological evidence of high-grade B-cell lymphoma or a documented history of an indolent lymphoproliferative disorder were excluded. Molecular testing for MYC, BCL2, and BCL6 rearrangements was not systematically performed in this retrospective cohort, as addressed in the Limitations. As a retrospective exploratory study, no *a priori* sample size calculation was performed, and all eligible patients during the study period were included. All patients were untreated and underwent clinical staging according to lymphoma diagnosis and treatment guidelines (19). Among the patients, 3 were classified as stage I, 5 as stage II, 9 as stage III, and 25 as stage IV.

This study involved human subjects and was conducted in accordance with local legislation and institutional requirements. Written informed consent for participation in this study was obtained from all participants.

### 2.1 PET/CT data acquisition

All patients underwent 18F-FDG PET/CT scans using a GE Discovery MI PET/CT scanner (GE Healthcare, USA). Before the examination, each patient was fasted for over 6 hours, and their blood glucose levels remained within the normal range. 18F-FDG (a dose of 0.10 to 0.12 mCi/kg) was intravenously injected based on the patient’s weight. Afterward, the patient was instructed to rest for 60 to 70 minutes and empty their bladder 5 minutes before the examination.

The CT scanning parameters were as follows:

- For the head: tube voltage of 120 kV, tube current of 150 mA, slice thickness of 3.75 mm.
- For the chest: tube voltage of 120 kV, tube current of 364 mA, slice thickness of 1.25 mm or 2.5 mm.
- For other body parts: tube voltage of 120 kV, tube current of 115 mA, slice thickness of 3.75 mm.

The PET scan was conducted in 3D mode. For the body, the scan range extended from the skull base to mid-femur, with 6 to 7 beds acquired based on the patient’s height using a scanning parameter of 2min/bed. For the head, the scan range was from the cranial vault to the skull base, with one bed acquired at a scanning parameter of 3min/bed. PET images were corrected for attenuation using the CT images and were reconstructed using the iterative algorithm (4 iterations, 24 subsets) and partial volume effect correction(PVC). The transverse, coronal, and sagittal views of PET, CT, and PET+CT fusion images, as well as maximum intensity projection (MIP) images, were obtained.

### 2.2 PET lesion delineation and metabolic quantitative analysis

All patients’ PET/CT DICOM data were loaded into the 3D Slicer platform (version 5.62, http://www.slicer.org). First, each patient’s PET image was co-registered with the CT image using the Elastix registration extension (20). Next, the whole-body CT images were segmented using the TotalSegmentation extension to obtain the whole-body organs (6). The organ segmentation was then copied to PET images to assist in the subsequent lesion segmentation. Third, the regions of interest (ROIs) for lesions were automatically delineated using a threshold of 41% maximum standard uptake value (SUVmax), which is recommended by the EANM procedure guidelines (6) and has been extensively validated in DLBCL cohorts (8, 9). Given the more recently advocated fixed SUV 4.0 threshold for metabolic tumor volume measurement (21, 22), the potential impact of threshold selection was additionally considered, as addressed in the Discussion. All PET/CT images were independently reviewed by two experienced nuclear medicine physicians, who manually removed benign lesions and physiological uptakes; any discrepancy in lesion inclusion between the two readers was resolved by consensus discussion, and all quantitative parameters were computed automatically on the final consensus lesion set. Finally, the conventional PET metabolic parameters, including SUVmax, mean standard uptake value(SUVmean), MTV, and TLG, were automatically calculated for all lesion ROIs.

In addition, four PET diffusion parameters of lesions were automatically measured for patients in stages II to IV who had more than two lesions. These parameters included:

- Dmax: the distance between the two furthest lesions.
- Dmaxbulk: the distance between the largest lesion and the furthest lesion from it.
- SPREADbulk: the sum of all distances from the largest lesion to all other lesions.
- SPREAD: the largest value, over all lesions, of the sum of the distances from a given lesion to all other lesions.

The starting and ending points of each measurement were the centers of each lesion. All Dmax, Dmaxbulk, and SPREAD values were corrected according to the body surface area (23).

### 2.3 PET image feature extraction

A total of 121 radiomics features were extracted for each lesion region of interest (ROI) using the Pyradiomics software (version 3.1.0). These radiomics features included 19 histogram features, 27 2D/3D shape features, and 75 texture features (24, 25). Specifically, the texture features comprised 24 GLCM (gray-level co-occurrence matrix) features, 16 GLRLM (gray-level run-length matrix) features, 16 GLSZM (gray-level size-zone matrix) features, 14 GLDM (gray-level dependence matrix) features, and 5 NGTDM (neighborhood gray-tone difference matrix) features.

Second, the radiomics features of lesions were normalized for each patient in stages II to IV using the Z-score method. Next, Pearson correlation coefficients were calculated for pairs of lesions. These pairwise inter-lesion Pearson correlation coefficients were then used to establish a lesion radiomics similarity matrix for each patient.

### 2.4 PET metabolic network construction and quantitative analysis

A lesion metabolic network for each patient in stages II to IV was constructed by defining the node as the lesion ROIs, and the edge as the calculated inter-lesion radiomics Pearson correlations. To explore the topological structure of the lesion metabolic networks, we used NetworkX open-source software (release 3.2.1, https://networkx.org) (26) to calculate various graph-theoretic parameters. These parameters included the adjacency matrix, mean degree (MD), average shortest path length (ASPL) between nodes, degree Pearson correlation coefficient (DPCC), and average clustering coefficient (ACC). The degree of a node refers to the number of adjacent nodes. The DPCC computes the degree assortativity of the network, which measures the similarity of connections in the network concerning the node degree. The ACC measures how close neighboring nodes are within the network.

### 2.5 Statistical analysis

All programs and statistical analyses were implemented on Python 3.9, R 4.2, and NetworkX 3.2.1. Continuous variables were presented as mean ± SD. The T-test method was used for the normal distributed continuous variable and the Mann-Whitney U test was utilized for the continuous variable with non-normal distribution. The nominal variables, shown as percentages, were analyzed by (corrected) chi-square test. The Shapiro–Wilk test was used to determine the normal distribution of data. The T-test for two groups, and analysis of variance (ANOVA) for more than two groups were employed for normally distributed continuous variables. A two-tailed p-value <0.05 indicated statistical significance. Effect sizes (Cohen’s f for multi-group comparisons and Cohen’s d for two-group comparisons) were calculated and are reported as descriptive measures only.

The two-tailed T-test was used to compare the common PET metabolic parameters, diffusion parameters, and lesion metabolic network parameters across different DLBCL staging groups.

**Figure 1:**
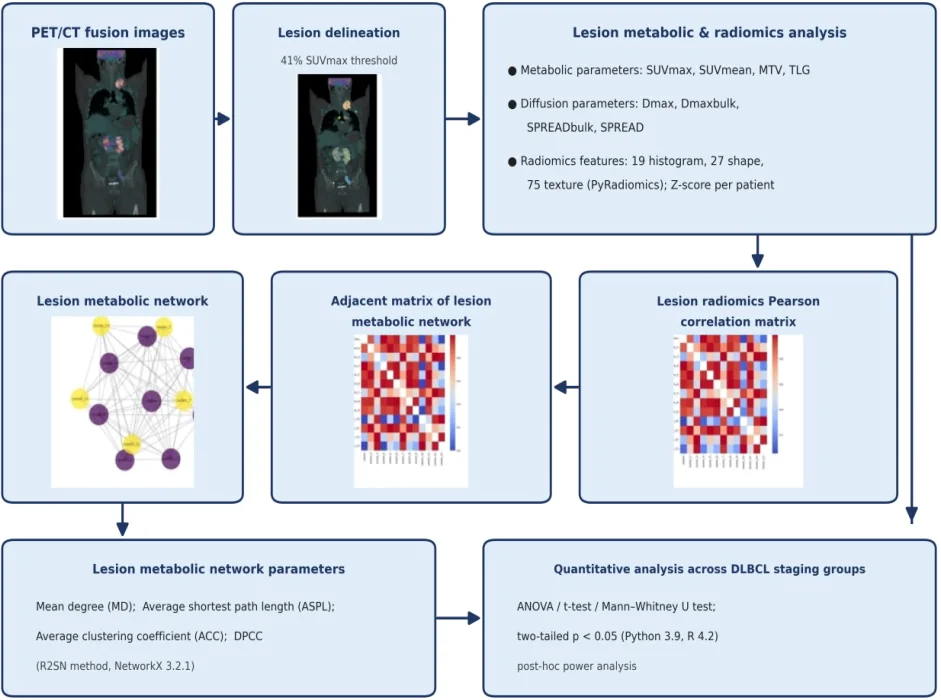
Schematic workflow of 18F-FDG PET/CT metabolic network analysis.

The workflow for analyzing 18F-FDG PET/CT metabolic networks was schematically presented in **Figure 1**.

## 3 Results

Among the 42 DLBCL patients enrolled, there were 23 males and 19 females, aged 61.19 ± 12.67 years, with a height and weight of 164.77 ± 8.51cm and 65.37 ± 14.21kg, respectively. All enrolled DLBCL patients underwent radiomics feature extraction and R2SN-based lesion metabolic network construction, and lymphoma diffusion parameters were calculated.

**Table 1** presents the results of quantitative analyses of lesion metabolic parameters, diffusion parameters, and metabolic network parameters across four staging groups for all 42 patients with DLBCL. Note that only those patients in stages II to IV had diffusion parameters and metabolic network parameters computed. As shown in **Table 1**, there were no significant differences in gender, age, height, or weight among the four stage groups (p > 0.5). Four metabolic parameters and four diffusion parameters, such as SUVmax, MTV, TLG, and Dmax, generally increased with higher stage values; however, no differences were observed among the four stage groups (p > 0.1). Within four metabolic network parameters, there were no differences in mean degree, ASPL, and ACC (p > 0.1). However, the DPCC showed highly significant differences among stages II to IV (p < 0.001). Moreover, the absolute value of DPCC decreased largely as the stage increased. This suggests that DPCC within the metabolic network is significant for staging DLBCL. The stage distribution of this cohort was imbalanced: stage IV cases predominated (n = 25), whereas stages I–III comprised only 3, 5, and 9 patients, and some subgroups were very small (for example, 2 females and 3 males in stage II). The corresponding subgroup estimates should therefore be interpreted with caution.

**Table 1:** Comparison of quantitative parameters across different stage groups in 42 patients with DLBCL.

Variables	Stage I N = 3	Stage II N = 5	Stage III N = 9	Stage IV N = 25	Statistics	P-value
Gender (Female)	1 (33.33%)	2 (40.00%)	4 (44.44%)	12 (48.00%)	–	1.00
Gender (Male)	2 (66.67%)	3 (60.00%)	5 (55.56%)	13 (52.00%)		
Age (years)	43.67 ± 17.24	57.00 ± 18.60	64.44 ± 9.95	63.40 ± 10.05	0.789	0.462
Height (cm)	170.00 ± 10.00	163.60 ± 4.98	166.22 ± 10.65	164.72 ± 8.76	0.158	0.855
Weight (Kg)	80.33 ± 16.17	69.20 ± 8.04	68.56 ± 17.09	63.02 ± 14.50	0.706	0.5
SUVmax	22.11 ± 4.74	23.86 ± 16.05	38.84 ± 17.38	35.19 ± 16.00	1.401	0.259
MTV	136.02 ± 159.45	184.67 ± 269.18	535.51 ± 569.22	451.41 ± 455.23	0.941	0.399
SUVmean	8.01 ± 1.59	6.59 ± 3.08	7.26 ± 2.44	6.40 ± 1.77	0.545	0.585
TLG	1346.25 ± 1710.67	1420.42 ± 2311.45	5843.64 ± 8455.07	3492.11 ± 3705.45	1.331	0.277
Dmax (mm)	–	293.78 ± 210.42	461.23 ± 142.01	487.61 ± 177.35	2.581	0.09
Dmaxbulk (mm)	–	255.98 ± 214.20	357.10 ± 108.63	382.78 ± 162.31	1.328	0.278
SPREADbulk (mm)	–	532.45 ± 448.66	1565.41 ± 958.40	3374.62 ± 3670.98	2.497	0.096
SPREAD (mm)	–	1371.45 ± 1370.43	12379.77 ± 12633.58	43253.82 ± 80454.35	1.294	0.287
Mean Degree	–	0.80 ± 0.20	0.75 ± 0.16	0.82 ± 0.10	0.941	0.4
ASPL	–	1.56 ± 0.91	1.59 ± 0.42	1.36 ± 0.26	1.275	0.292
DPCC	–	-0.40 ± 0.15	-0.17 ± 0.14	-0.11 ± 0.06	19.645	< 0.001
ACC	–	0.77 ± 0.27	0.71 ± 0.19	0.80 ± 0.13	1.045	0.362

SUVmax:maximum standard uptake value; MTV, metabolic tumor volume; SUVmean, mean standard uptake value; TLG, total lesion glycolysis; Dmax, the largest distance between two lesions; Dmaxbulk, the distance between the largest lesion and the lesion farthest from that bulk; SPREADbulk, the sum of the distances of the bulky lesion from all other lesions; SPREAD, the largest value, over all lesions, of the sum of the distances from a lesion to all the others; ASPL, average shortest path length; DPCC, degree Pearson correlation coefficient; ACC, average clustering coefficient.

We combined stages I and II, and stages III and IV respectively. **Table 2**, **3** present the comparison results between these groups. Unlike **Table 1**, there were significant differences in age (p < 0.05), SUVmax (p < 0.05) and Dmax (p < 0.05). Notably, the DPCC still showed a highly significant difference (p < 0.001). Because the diffusion and metabolic network parameters cannot be computed in single-lesion stage I cases, the two-group comparisons were performed separately: conventional metabolic parameters were compared between stages I+II (n=8) and stages III+IV (n=34) (**Table 2**), whereas diffusion and metabolic network parameters were compared between stage II (n=5) and stages III+IV (n=34) (**Table 3**). The effect size for DPCC was large (Cohen’s f = 0.99 for the three-stage comparison; Cohen’s d = 2.72 for the two-group comparison); as this was a retrospective exploratory study without *a priori* sample size calculation, these effect sizes are reported as descriptive measures only.

**Table 2:** Comparison of conventional metabolic parameters between DLBCL stages I+II and stages III+IV.

Variable	Stage I+II N = 8	Stage III+IV N = 34	Statistics	P-value
Gender (Female)	3 (37.50%)	16 (47.06%)	0.0	1.0
Gender (Male)	5 (62.50%)	18 (52.94%)		
Age (years)	51.56 ± 17.05	63.68 ± 9.88	-2.78	0.008
Height (cm)	165.11 ± 7.36	165.12 ± 9.15	-0.002	0.998
Weight (Kg)	72.00 ± 11.99	64.49 ± 15.16	1.373	0.177
SUVmax	24.26 ± 12.05	36.16 ± 16.19	-2.052	0.047
MTV	175.50 ± 209.48	473.67 ± 480.29	-1.805	0.078
SUVmean	7.71 ± 2.99	6.63 ± 1.97	1.311	0.197
TLG	1579.07 ± 1928.00	4114.57 ± 5331.52	-1.392	0.171

SUVmax, maximum standard uptake value; MTV, metabolic tumor volume; SUVmean, mean standard uptake value; TLG, total lesion glycolysis.

**Table 3:** Comparison of diffusion and metabolic network parameters between DLBCL stage II and stages III+IV.

Variable	Stage II N = 5	Stage III+IV N = 34	Statistics	P-value
Dmax (mm)	293.78 ± 210.42	480.63 ± 167.04	-2.265	0.029
Dmaxbulk (mm)	255.98 ± 214.20	375.98 ± 148.83	-1.594	0.12
SPREADbulk (mm)	532.45 ± 448.66	2895.71 ± 3268.00	-1.597	0.119
SPREAD (mm)	1371.45 ± 1370.43	35081.28 ± 70266.70	-1.061	0.296
Mean Degree	0.80 ± 0.20	0.80 ± 0.12	-0.131	0.896
ASPL	1.56 ± 0.91	1.42 ± 0.32	0.699	0.489
DPCC	-0.40 ± 0.15	-0.12 ± 0.09	-5.957	< 0.001
ACC	0.77 ± 0.27	0.77 ± 0.15	-0.12	0.905

Dmax, the largest distance between two lesions; Dmaxbulk, the distance between the largest lesion and the lesion farthest from that bulk; SPREADbulk, the sum of the distances of the bulky lesion from all other lesions; SPREAD, the largest value, over all lesions, of the sum of the distances from a lesion to all the others; ASPL, average shortest path length; DPCC, degree Pearson correlation coefficient; ACC, average clustering coefficient.

Diffusion and metabolic network parameters cannot be computed in single-lesion stage I cases; therefore, the early-stage group in this table comprises the five stage II patients.

## 4 Discussion

DLBCL is a highly heterogeneous type of lymphoma that constitutes a large portion of lymphoma cases (27). Accurate staging is crucial for effective treatment planning and prognosis. The total metabolic tumor volume and diffusion parameters between lesions measured based on 18F-FDG PET/CT images have become important biomarkers for assessing prognosis and treatment efficacy in DLBCL (9, 10). However, these biomarkers are rarely applied in staging. While the structure or metabolic network has been used successfully in brain neuroscience studies, it has not been widely explored for understanding the interconnections between lesions in DLBCL.

In this study, we analyzed and compared conventional FDG PET/CT metabolic parameters (such as SUV and MTV), diffusion parameters (such as Dmax and SPREAD), and metabolic network parameters (such as DPCC and ACC), for DLBCL stages I to IV. The results showed that DPCC of the FDG PET/CT metabolic network exhibited a highly significant difference among stages II to IV (p < 0.001) and between stage II and stages III+IV (p < 0.001). In contrast, SUVmax and Dmax exhibited significance (p < 0.05) only in the two-group comparisons (**Table 2** and **3**). Other parameters, such as SUVmean, MTV, ACC, etc., did not show significant differences (p > 0.05) among the various stage groups. These findings suggest that DPCC, Dmax, and SUVmax from FDG PET/CT are associated with clinical DLBCL staging, with DPCC showing the strongest stage-related association in this exploratory cohort. Because no receiver operating characteristic analysis or formal model comparison was performed, no claim of superior discriminative performance can be made.

Notably, the absolute value of DPCC decreased as the stage increased. This indicates that as the DLBCL progresses to higher stages, the strength of the degree’s Pearson correlation decreases. It suggests that the heterogeneity of DLBCL increases significantly with the higher stages (28). Additionally, we observed that the DPCC values across all stage groups were negative, which means the DLBCL metabolic networks are disassortative. In other words, lesion nodes with a high degree tend to connect to lesions with a low degree in DLBCL metabolic networks. This architecture can be interpreted biologically (29). In lower-stage disease, the metabolic network typically comprises only a few lesions: one or several metabolically dominant “hub” lesions are connected to smaller satellite lesions, forming a hub-and-spoke topology that yields a strongly negative DPCC. As the disease progresses to higher stages, the number of lesions increases and their radiomics phenotypes become more diverse, connections increasingly form between lesions of similar degree, the degree correlation weakens, and the absolute value of DPCC decreases accordingly. The progressive decline of |DPCC| from stage II (−0.40) through stage III (−0.17) to stage IV (−0.11) therefore quantitatively mirrors the increasing inter-lesion metabolic heterogeneity that accompanies systemic dissemination, which substantiates why DPCC carries staging information beyond conventional metabolic and diffusion parameters. In graph theory, assortativity and disassortativity provide insights into the structure of a network, as well as its dynamic behaviors and robustness (29). However, utilizing DPCC to analyze heterogeneity changes across different stages will require more data and in-depth biological investigation.

The 18F-FDG PET/CT metabolic network is a novel approach for analyzing tumors, particularly their interconnections (16). This method complements conventional metabolic and diffusion analyses (17). In this study, we constructed a metabolic network by utilizing a variety of radiomics features, including first-order, second-order, and shape features of tumors. In comparison to conventional metabolic parameters like SUV, as well as diffusion parameters like Dmax, which can be viewed as one-dimension and two-dimension measurements, the quantitative parameters of the metabolic network can be considered as high-dimension measurements. They can provide more comprehensive information about lesions or tumors. Consequently, the inter-group DPCC differences were more pronounced than those of Dmax or SUVmax in this exploratory cohort. Despite previous research on metabolic brain networks in lymphoma (16),the R2SNs method is the latest feature extraction method, which extracts more radiomics parameters to construct brain networks than Kullback Leibler divergence based similarity (KLS).

Regarding lesion delineation, the 41% SUVmax relative threshold used in this study is recommended by the EANM procedure guidelines (6) and remains widely used in lymphoma studies (8, 9), although more recent investigations advocate a fixed SUV 4.0 threshold for measuring baseline metabolic tumor volume in DLBCL (21, 22). The delineation threshold may affect lesion boundaries, radiomics feature values, pairwise inter-lesion correlations, and the resulting network topology; because no sensitivity analysis with the fixed SUV 4.0 threshold was performed, the robustness of DPCC across segmentation methods remains unclear. A formal head-to-head comparison between the 41% SUVmax and the fixed SUV 4.0 thresholds is therefore warranted in future validation studies.

Recent work has demonstrated that integrating 18F-FDG PET radiomics with circulating tumor DNA (ctDNA) liquid biopsy improves outcome prediction in newly diagnosed DLBCL (30). Metabolic network connectivity and ctDNA capture complementary dimensions of tumor heterogeneity—spatial-metabolic and genomic, respectively. Therefore, integrating PET/CT metabolic network connectivity with ctDNA liquid biopsy at the time of DLBCL diagnosis represents a promising future perspective for further improving staging accuracy and prognostication, and we plan to explore this integration in subsequent prospective studies.

Technical advances in PET instrumentation may further strengthen this approach. New-generation digital PET/CT systems equipped with silicon photomultiplier (SiPM) detectors provide substantially improved spatial resolution, time-of-flight resolution, and sensitivity compared with conventional photomultiplier-based systems (31), and long axial field-of-view (LAFOV) scanners offer an order-of-magnitude higher effective sensitivity, enabling superior small-lesion detectability, shorter acquisition times, and reduced injected activities (32). These developments are expected to improve the accuracy of lesion delineation and radiomics quantification, and thereby the robustness of metabolic network connectivity analysis in future studies.

While we identified DPCC as well as Dmax and SUVmax as potential biomarkers associated with clinical staging of DLBCL, several limitations of this study should be noted. First, this was a retrospective exploratory study with a small sample size recruited from a single center; no *a priori* sample size calculation was performed, and all eligible patients during the study period were included. Second, the stage distribution was imbalanced, with a predominance of stage IV cases (25/42) and very small numbers in stages I–III; this imbalance limits the precision of subgroup estimates (for example, gender distribution within stage II) and the generalizability of the findings. Third, the diffusion and metabolic network parameters cannot be computed in single-lesion stage I cases, so the early-stage comparator for these parameters comprised only five stage II patients. Fourth, no independent external validation cohort was available. Fifth, the 41% SUVmax delineation threshold was not formally compared with the more recently recommended fixed SUV 4.0 threshold, and the robustness of DPCC across segmentation methods remains unclear. Sixth, molecular testing for MYC, BCL2, and BCL6 rearrangements and molecular subtyping were not systematically performed in this retrospective cohort; the diagnosis of *de novo* DLBCL, NOS, was based on the available histopathological and immunohistochemical data. Seventh, given the limited sample size and the exploratory design, we did not assess the value of metabolic network connectivity for predicting clinical outcomes (such as progression-free or overall survival) in this series; this important question requires dedicated analysis in larger cohorts with mature follow-up. We plan to collect more data, especially from multiple centers, for a more comprehensive analysis. In addition, this study only focused on DLBCL. The clinical value of metabolic network analysis for staging other types of lymphoma and evaluating the treatment efficacy still needs to be explored further.

## 5 Conclusions

In DLBCL patients with multiple measurable lesions (stages II–IV), the DPCC, a quantitative indicator of PET radiomics-based metabolic networks, was associated with clinical stage and may provide complementary network-level information beyond conventional metabolic and diffusion parameters. These exploratory findings require validation in larger, multicenter prospective cohorts.

## Data Availability

The data supporting the findings of this study are not publicly available due to patient privacy considerations. Data requests may be directed to the corresponding author and will be considered in accordance with applicable institutional and ethical requirements.

## References

[1] Shi Y Xu Y Shen H Jin J Tong H Xie W . Advances in biology, diagnosis and treatment of DLBCL. Ann Hematol. (2024) 103:3315–34. doi: 10.1007/s00277-024-05880-z 39017945 PMC11358236

[2] Li S Young KH Medeiros LJ . Diffuse large B-cell lymphoma. Pathology. (2018) 50:74–87. doi: 10.1016/j.pathol.2017.09.006 29167021

[3] Lodhi N Tun M Nagpal P Inamdar AA Ayoub NM Siyam N . Biomarkers and novel therapeutic approaches for diffuse large B-cell lymphoma in the era of precision medicine. Oncotarget. (2020) 11:4045–73. doi: 10.18632/oncotarget.27785 33216822 PMC7646825

[4] Huang C Hu H Zheng X . Application effect of 18F-FDG PET/CT technique in diagnosis and prognosis evaluation of lymphoma. SLAS Technol. (2024) 29:100176. doi: 10.1016/j.slast.2024.100176 39151752

[5] Boellaard R Delgado-Bolton R Oyen WJG Giammarile F Tatsch K Eschner W . FDG PET/CT: EANM procedure guidelines for tumour imaging: version 2.0. Eur J Nucl Med Mol Imaging. (2015) 42:328–354. doi: 10.1007/s00259-014-2961-x PMC4315529 25452219

[6] Wasserthal J Breit HC Meyer MT Pradella M Hinck D Sauter AW . TotalSegmentator: robust segmentation of 104 anatomic structures in CT images. Radiol Artif Intell. (2023) 5:e230024. doi: 10.1148/ryai.230024 37795137 PMC10546353

[7] Gupta N Singh N . To determine the prognostic significance of 18F-fluorodeoxyglucose positron emission tomography/computed tomography scan-derived parameters (total lesion glycolysis and metabolic tumor volume) in patients of diffuse large B-cell lymphoma with only nodal involvement. Indian J Nucl Med. (2020) 35:100–4. doi: 10.4103/ijnm.IJNM_6_20 32351262 PMC7182330

[8] Jo JH Chung HW Kim SY Lee MH So Y . FDG PET/CT maximum tumor dissemination to predict recurrence in patients with diffuse large B-cell lymphoma. Nucl Med Mol Imaging. (2023) 57:26–33. doi: 10.1007/s13139-022-00782-2 36643943 PMC9832207

[9] Cottereau AS Nioche C Dirand AS Clerc J Morschhauser F Casasnovas O . 18F-FDG PET dissemination features in diffuse large B-cell lymphoma are predictive of outcome. J Nucl Med. (2020) 61:40–5. doi: 10.2967/jnumed.119.229450 PMC6954460 31201248

[10] Girum KB Rebaud L Cottereau AS Meignan M Clerc J Vercellino L . 18F-FDG PET maximum-intensity projections and artificial intelligence: a win-win combination to easily measure prognostic biomarkers in DLBCL patients. J Nucl Med. (2022) 63:1925–32. doi: 10.2967/jnumed.121.263501 35710733 PMC9730929

[11] Guerra L Albano D Angelillo P Di Rocco A Farina M Farolfi A . PET-guided early identification of CAR-T candidates in diffuse large B-cell lymphoma: a multidisciplinary expert consensus. Eur J Nucl Med Mol Imaging. (2026). doi: 10.1007/s00259-026-07999-9 42360330 PMC13633147

[12] Lu W Song T Li J Zhang Y Lu J . Individual-specific network based on 18F-FDG PET aberrant multi-level metabolic metabolisms in Parkinson's disease. Hum Brain Mapp. (2024) 45:e70026. doi: 10.1002/hbm.70026 39300894 PMC11413412

[13] Reed MB Ponce de León M Vraka C Rausch I Godbersen GM Popper V . Whole-body metabolic functional connectivity framework with PET. NeuroImage. (2023) 271:120030. doi: 10.1016/j.neuroimage.2023.120030 36925087

[14] Zhao K Zheng Q Che T Dyrba M Li Q Ding Y . Regional radiomics similarity networks (R2SNs) in the human brain: reproducibility, small-world properties and a biological basis. Netw Neurosci. (2021) 5:783–97. doi: 10.1162/netn_a_00200 34746627 PMC8567836

[15] Goldman AW Burmeister Y Cesnulevicius K Herbert M Kane M Lescheid D . Bioregulatory systems medicine: an innovative approach to integrating the science of molecular networks, inflammation, and systems biology with the patient's autoregulatory capacity? Front Physiol. (2015) 6:225. doi: 10.3389/fphys.2015.00225 26347656 PMC4541032

[16] Yu J Hua L Cao X Chen Q Zeng X Yuan Z . Construction of an individualized brain metabolic network in patients with advanced non-small cell lung cancer by the Kullback–Leibler divergence-based similarity method: a study based on 18F-fluorodeoxyglucose positron emission tomography. Front Oncol. (2023) 13:1098748. doi: 10.3389/fonc.2023.1098748 36969017 PMC10036828

[17] Liu J Tang M Zhu D Ruan G Zou S Cheng Z . The remodeling of metabolic brain pattern in patients with extracranial diffuse large B-cell lymphoma. EJNMMI Res. (2023) 13:94. doi: 10.1186/s13550-023-01046-6 37902852 PMC10616001

[18] Alaggio R Amador C Anagnostopoulos I Attygalle AD Araujo IBO Berti E . The 5th edition of the World Health Organization classification of haematolymphoid tumours: lymphoid neoplasms. Leukemia. (2022) 36:1720–48. doi: 10.1038/s41375-022-01620-2 35732829 PMC9214472

[19] Yoo KH . Staging and response assessment of lymphoma: a brief review of the Lugano classification and the role of FDG PET/CT. Blood Res. (2022) 57:75–8. doi: 10.5045/br.2022.2022055 35483930 PMC9057662

[20] Klein S Staring M Murphy K Viergever MA Pluim JP . Elastix: a toolbox for intensity-based medical image registration. IEEE Trans Med Imaging. (2010) 29:196–205. doi: 10.1109/TMI.2009.2035616 19923044

[21] Ilyas H Mikhaeel NG Dunn JT Rahman F Moloney P Barrington SF . Defining the optimal method for measuring baseline metabolic tumour volume in diffuse large B cell lymphoma. Eur J Nucl Med Mol Imaging. (2018) 45:1142–54. doi: 10.1007/s00259-018-3953-z 29460024 PMC5953976

[22] Mikhaeel NG Heymans MW Eertink JJ de Vet HCW Boellaard R Dührsen U . Proposed new dynamic prognostic index for diffuse large B-cell lymphoma: international metabolic prognostic index. J Clin Oncol. (2022) 40:2352–60. doi: 10.1200/JCO.21.02063 35357901 PMC9287279

[23] Beichel RR Van Tol M Ulrich EJ Bauer C Chang T Plichta KA . Semiautomated segmentation of head and neck cancers in 18F-FDG PET scans: a just-enough-interaction approach. Med Phys. (2016) 43:2948–64. doi: 10.1118/1.4948679 27277044 PMC4874930

[24] van Griethuysen JJM Fedorov A Parmar C Hosny A Aucoin N Narayan V . Computational radiomics system to decode the radiographic phenotype. Cancer Res. (2017) 77:e104–7. doi: 10.1158/0008-5472.CAN-17-0339 29092951 PMC5672828

[25] Su GH Xiao Y You C Zheng RC Zhao S Sun SY . Radiogenomic-based multiphasic imaging heterogeneity analysis and therapeutic targets. Sci Adv. (2023) 9:eadf0837. doi: 10.1126/sciadv.adf0837 37801493 PMC10558123

[26] Hagberg AA Schult DA Swart PJ . (2008). “ Exploring network structure, dynamics, and function using NetworkX”, in: Proceedings of the 7th Python in Science Conference (SciPy 2008) (Pasadena, CA: SciPy), 11–5.

[27] Sheng LS Shen R Yan ZX Wang C Zheng X Zhang YL . 18F-FDG PET/CT metrics-based stratification of large B-cell lymphoma receiving CAR-T cell therapy: immunosuppressive tumor microenvironment as a negative prognostic indicator in patients with high tumor burden. biomark Res. (2024) 12:104. doi: 10.1186/s40364-024-00650-5 39272132 PMC11401356

[28] Al Tabaa Y Bailly C Kanoun S . FDG-PET/CT in lymphoma: where do we go now? Cancers (Basel). (2021) 13:5222. doi: 10.3390/cancers13205222 34680370 PMC8533807

[29] Newman MEJ . Assortative mixing in networks. Phys Rev Lett. (2002) 89:208701. doi: 10.1103/PhysRevLett.89.208701 12443515

[30] Dondolin R Garrou F Almasri M Terzi Di Bergamo L Cosentino C Bruscaggin A . Integration of [18F]FDG-PET radiomics with liquid biopsy improves outcome prediction in newly diagnosed diffuse large B-cell lymphoma. Leukemia. (2025) 39:2207–14. doi: 10.1038/s41375-025-02688-2 40629066 PMC12380602

[31] van Sluis J de Jong J Schaar J Noordzij W van Snick P Dierckx R . Performance characteristics of the digital Biograph Vision PET/CT system. J Nucl Med. (2019) 60:1031–6. doi: 10.2967/jnumed.118.215418 30630944

[32] Alberts I Hünermund JN Prenosil G Mingels C Bohn KP Viscione M . Clinical performance of long axial field of view PET/CT: a head-to-head intra-individual comparison of the Biograph Vision Quadra with the Biograph Vision PET/CT. Eur J Nucl Med Mol Imaging. (2021) 48:2395–404. doi: 10.1007/s00259-021-05282-7 33797596 PMC8241747

